# Risk Factors for Surgical Complications in Ventral Hernia Repair

**DOI:** 10.1007/s00268-018-4642-6

**Published:** 2018-04-26

**Authors:** Mikael Lindmark, Karin Strigård, Thyra Löwenmark, Ursula Dahlstrand, Ulf Gunnarsson

**Affiliations:** 1 Department of Surgical and Perioperative Sciences, Umeå University, 901 87 Umeå, Sweden; 2 Department of Clinical Science, Intervention and Technology, Karolinska Institutet, 141 86 Stockholm, Sweden; 3 Centre for Digestive Diseases, Karolinska University Hospital, 171 76 Stockholm, Sweden

## Abstract

**Background:**

The aim of this study was to identify risk factors for an adverse event, i.e. early surgical complication, need for ICU care and readmission, following ventral hernia repair. Our hypothesis was that there is an association between an increased complication rate following ventral hernia repair and specific factors, including hernia size, BMI > 35, concomitant bowel surgery, ASA-class, age, gender and method of hernia repair.

**Methods:**

Data from a hernia database with prospectively entered data on 408 patients operated for ventral hernia between 2007 and 2014 at two Swedish university hospitals were analysed. A 3-month follow-up of complications, need for intensive care and readmission, was performed by reviewing the medical records.

**Results:**

Eighty-one of 408 patients (20%) had a registered complication. Fifty-eight (14%) of these were classed as Clavien I–IIIa, and in 19 cases a Clavien IIIb–IV complication was reported. Large hernia size was associated with increased risk for early complication. A Kendall Tau test analysis revealed a proportional relationship between hernia size and modified Clavien outcome class (*p* < 0.001). Morbid obesity, ASA-class, method, hernia recurrence, age and concomitant bowel surgery were not statistically significant predictors of adverse events.

**Conclusions:**

Assessment of hernia aperture size is of great importance in the preoperative evaluation of ventral hernia patients to consider risk for post-operative complications. These results suggest a careful attitude when applying watchful waiting concepts and when postponing hernia surgery to achieve weight loss. A delaying attitude may result in increased risk of complications caused by increasing hernia size.

## Introduction

Incisional hernia is a common complication after abdominal surgery and occurs in approximately 10–25% of patients undergoing laparotomy [1, 2]. There are several factors affecting the risk of incisional hernia development. Age, obesity, infection, immunomodulating therapy, diabetes and smoking are well-known factors. Several choices made by the surgeon can also affect the occurrence of incisional hernia: suture technique (small bites), suture material and location of the incision [3–5]. Up to 30% of these hernias require repair since incisional hernia is an important cause of morbidity [2]. At present, there is insufficient Grade A data to provide evidence-based recommendations regarding key steps in the treatment algorithm of ventral hernia. This hampers risk–benefit analysis when evaluating the patient prior to ventral hernia repair, and management is largely based on the preference of the surgeon.

For more than a decade now, open mesh hernia repair has become the standard procedure for ventral hernia repair. Many surgeons consider sublay repair to be the gold standard for incisional hernia repair. In a recent meta-analysis of studies comparing sublay to onlay repair, however, only surgical site infection (SSI) rate differed, with a lower frequency in sublay repair [6]. No differences in recurrence rate were seen, though there was a trend towards more recurrences in the onlay group, and seroma formation was equally distributed between the two groups [6]. Laparoscopic ventral hernia repair (LVHR) is increasing in popularity, and short-term results of studies comparing SSI rates between LVHR and open techniques are promising [2]. More research comparing ventral hernia repair techniques is required, especially long-term follow-up studies, in view of the risk for material-related complications associated with the intraperitoneal onlay mesh technique (IPOM), where mesh is in close contact with the intestine [7].

It is known that more complex incisional hernia repairs are associated with a high complication rate [7]. Complication rates following incisional hernia repair vary considerably, and rates as high as 50% are not uncommon when treating giant incisional hernias [8]. Open techniques predispose to complications related to the wound, often called surgical site occurrence (SSO), including seroma, haematoma and wound healing disturbances. Introduction of the laparoscopic technique has resulted in a decrease in SSOs and length of hospital stay [9]. Theoretically, the laparoscopic technique exposes the patient to a different risk spectrum due to mandatory breach of the peritoneum, and mesh might come into direct contact with viscera. Some studies indicate a higher risk for undetected enterotomy [10]. Bowel injury seems to be correlated with the presence of dense adhesions. Little literature exists concerning the risk profile of laparoscopic ventral hernia repair, which is why there is a need of further studies [10].

Ventral hernia repair is one of the most common surgical procedures in the world. There is no universally accepted system of classification, which makes comparison between different studies difficult. Due to the high complexity of incisional hernia and the considerable variability in surgical management, we need to tailor our approach to match the individual patient.

The aim of this study was to identify risk factors for early complications following ventral hernia repair.

Our hypothesis was that there are specific risk factors that increase the complication rate in ventral hernia repair. The factors considered were: hernia size, BMI > 35 (*obesity class II)*, concomitant bowel surgery, ASA-class, age, gender, smoking and method of hernia repair.

## Materials and methods

Between 2007 and 2014, the University Hospital of Umeå (NUS) and the Karolinska University Hospital (K) had a common ventral hernia database with prospectively entered data. The database included both primary ventral hernias and incisional hernias. This database was created 2 years before the European Hernia Society launched their proposal for classification of primary and incisional abdominal wall hernias. Hence, it does not adhere to these classifications.

The registry was routinely used for quality assurance and monitoring of surgical production, but was also designed for research purposes. K served as a tertiary referral centre until 2013. Surgery at the NUS comprised mainly routine procedures, but there were also more complex cases of tertiary referral centre nature.

The database contained a detailed record of patient characteristics and hernia classification (see Appendix 1). A preoperative CT scan was not mandatory, wherefore hernia volumetry or determination of loss of domain was not an option in present analysis. However, surgeons were obliged to register the transverse measurement of the hernia aperture. Hernia size was documented during surgery [11].

Hernia was divided into midline and lateral hernias. Lateral hernia included incisional hernia after appendectomy, cholecystectomy, Puestow incision, flank incision and port-site hernias. Follow-up of complications within 3 months after surgery, need for intensive care and readmission, was performed by reviewing the medical records for both in- and outpatient clinic visits as well as visits at emergency ward. A research nurse supervised the quality and completeness of this process. Follow-up at 3 months was chosen to assure detection of post-operative surgical complications related to the hernia repair. Complications registered for the purposes of the study were: bleeding, infection, skin necrosis, fistula, bowel leakage, seroma, abscess, fistula without bowel connection, unplanned admission to the ICU, unplanned readmission and other complication (Table 1).

**Table 1:** Recorded surgical complications

Surgical complications
Bleeding
Infection
Skin necrosis
Fistula
Bowel leakage
Seroma
Abscess
Fistula without bowel connection
Unplanned admission to the ICU
Unplanned readmission
Other complication

### Patients

**Fig. 1:**
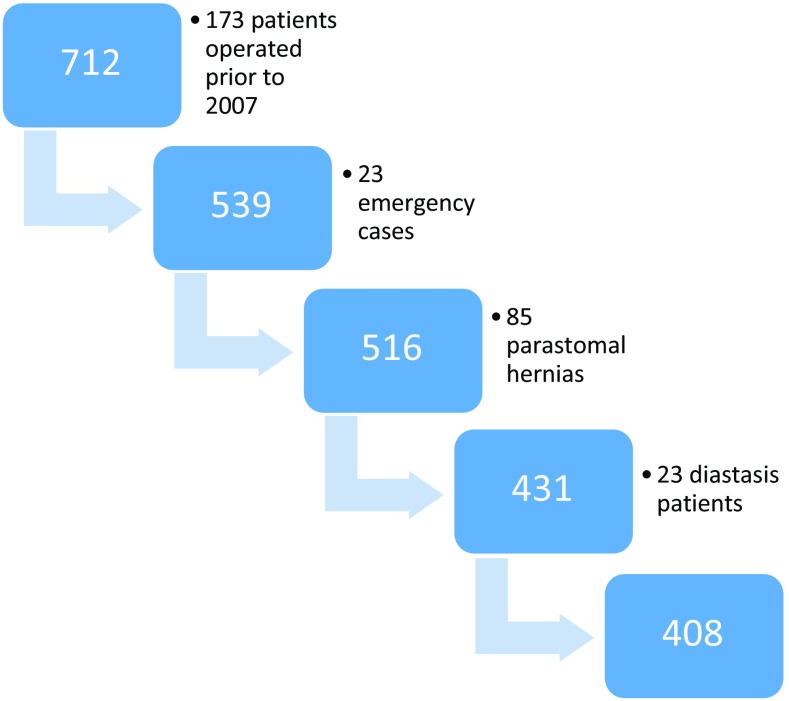
Inclusion and exclusions giving a total of 408 patients for final analysis

The study database covered a total of 712 patients with a primary ventral hernia or incisional hernia operated between 2007 and 2014. Parastomal hernias, diastasis and emergency procedures were excluded leaving 408 patients for analysis (Fig. 1). Since follow-up was limited to 3 months, all 408 cases included were eligible for analysis. Due to mandatory smoking cessation prior to benign surgery, introduced in several Swedish counties during the period of the study, few active smokers were accepted for hernia repair: 25 at K and 11 at NUS. Most smokers accepted for surgery either had severe symptoms or did not admit to smoking before being scheduled for repair.

Emergency cases were too few to provide power enough for inclusion in multivariate analyses; they were thus excluded. The management of parastomal hernia and diastasis differs from that of ventral hernia, and these cases were also excluded.

Skin graft repair is a method where an onlay repair is performed using autologous full-thickness skin as implant [12]. Except for the skin graft repairs, synthetic meshes were used in almost all repairs. Only in three cases a biologic patch was chosen.

The study was approved by the Regional Ethics Review Board in Stockholm (Dnr 2012/1961-31/1).

## Statistical methods

Patient data were registered in Access^®^ (Microsoft Corp.). The two hospitals, K and NUS, kept separate copies of the database, and patients were registered at the hospital where surgery was performed. Data were exported to, and all analyses performed in Statistica version 12 (StatSoft, Tulsa, OK, USA). Nonparametric analyses were generally used.

Complications were sorted into groups according to the Clavien–Dindo [13] classification of surgical complications, where Clavien classes I–IIIA were fused into one group representing patients with a complication not requiring surgical intervention, but including those requiring interventional radiological treatment. Clavien I–IIIA complications are considered to be mild to moderate complications, while Clavien IIIB (surgical intervention required), Clavien IV (admission to ICU) and Clavien V (death) were considered serious complications. An analysis of complication rates was performed.

Ordinal regression analyses were performed with surgical complication and logistic regression with readmission as dependent variables, and factors potentially influencing risk for complication as independent variables.

Potential pre-repair risk factors for surgical complications were: severe obesity (BMI ≥ 35 kg/m^2^), age, ASA-class, hernia size (transverse mm), method of repair, operation for recurrence, concomitant bowel surgery, gender and smoking. Hernia size and age were calculated as continuous variables. ASA-classes were dichotomised into two groups, ASA 1–2 and ASA 3–4. Significant risk factors in the univariate model were entered into a multivariate model.

The relationship between hernia aperture size and risk for surgical complication, according to a modified Clavien classification, was investigated using the Kendall Tau test.

## Results

Demographic data are shown in Table 2. Among the 408 patients analysed, 250 (61%) were female and 158 (39%) were male. Median BMI was 29 kg/m^2^. As seen, 201 (49%) of the patients were ASA-class 2 and 139 (34%) were ASA-class 3. Most of the hernias emanated from the midline 363 (88%). Fifty-three (12%) were lateral hernias (some patients had both midline and lateral hernias leading to total more than 408). Among the lateral hernias at K, 57% (20/35) were treated laparoscopically, while only two of eighteen lateral hernia repairs at NUS were treated laparoscopically. One patient operated for lateral hernia at K had a complication and none at NUS. The patient at K had an open IPOM procedure for a recurrent flank incisional hernia and was readmitted, but had no other reported complication. The number of operations was unevenly distributed between the participating hospitals; 277 (68%) were operated at K and 131 (32%) at NUS.

**Table 2:** Basic demographic data

Patients	NUS	*K*	Total
Total	131 (32.1%)	277 (67.9%)	408
Male gender	57 (43.5%)	101 (36.5%)	158 (38.7%)
Female	74 (56.5%)	176 (63.5%)	250 (61.3%)
ASA			
1	19 (14.5%)	41 (14.8%)	60 (14.7%)
2	74 (56.5%)	127 (45.8%)	201 (49.3%)
3	36 (27.5%)	103 (37.2%)	139 (34.1%)
4	1 (0.8%)	5 (1.8%)	6 (1.4%)
Missing	1 (0.7%)	1 (0.4%)	2 (0.5%)
BMI median (range)	29.4 (16.2–67.0)	28.4 (15.5–52.5)	28.7 (15.5–67.0)
Hernia size (mm)	60 (10–300)	80 (10–300)	70 (10–300)
Smoker, yes	11 (8.0%)	25 (9.0%)	36 (8.8%)
Age	61.9 (15.2–85.9)	59.7 (26.8–90.8)	60.5 (15.2–90.8)

408 patients operated for ventral hernia at the University Hospital of Umeå (NUS) and the Karolinska University Hospital (*K*). Measurements are shown with median and range. Hernia size is the transverse measurement in mm

The most common repair procedure was sublay mesh repair 212 (52%). Among the 97 (24%) IPOM procedures, there were 62 open and 35 laparoscopic repairs. At NUS, only three laparoscopic procedures were performed during the registration period. Forty-two (10%) onlay mesh repairs and 34 (8%) “other hernia repairs” were performed. The category “other hernia repairs” included suture repairs and cases operated with a combination of surgical methods (Table 3).

**Table 3:** Method of repair

	*N* patients NUS (%)	*N* patients *K* (%)	Total number of patients (%)
Sublay	62 (47%)	150 (54%)	212 (52%)
Onlay	12 (9%)	30 (11%)	42 (10%)
IPOM^a^	37 (28%)	60 (22%)	97 (24%)
Skin	4 (3%)	19 (7%)	23 (6%)
Other	16 (13%)	18 (6%)	34 (8%)

Number of patients and percentage of the entire group. Skin is autolog full-thickness transplants which are used as onlay repair. “Other” includes combination of methods and suture repair. NUS = University Hospital of Umeå, *K* = Karolinska University Hospital

^a^Intra-peritoneal onlay mesh

Thirty-one patients (8%) of total 408 were readmitted within the 3-month follow-up period. Of these, seven had had concomitant bowel surgery and seven had repair of a recurrent hernia. Seventeen sublay repairs and three onlay repairs were readmitted, while four of the readmitted patients had had an IPOM repair.

All repairs with concomitant bowel surgery were procedures that included opening of the bowel. In total, 52 (13%) patients (34 K, 18 NUS) had concomitant bowel surgery. Thirteen (25%) of these had at least one surgical complication registered. Most complications were classified as mild, such as wound infections, seromas and skin necrosis. There was one enteric leakage in K and two at NUS. Six (12%) of the patients with concomitant bowel surgery had a planned or unplanned visit at the ICU, and in five cases (10%) a reoperation was performed.

Eighty-one patients (20%) suffered a complication; 58 (14%) were classified as Clavien classes I–III A. The Clavien categories we used and associated frequencies are shown in Table 4. Twenty-one of 408 (5%) patients had an infection. Seven of the 42 (17%) onlay repair patients had a severe complication (Clavien >IIIA), these being two anastomotic leakages, one enterocutaneous fistula, three cases of skin necrosis and two infections. Four of these had had major concomitant bowel surgery followed by hernia repair at the same session. Three were colorectal procedures and one gastric sleeve too gastric bypass conversion. None of the onlay repairs with a severe complication was for recurrence or re-recurrence. Sixteen patients (20%) operated with a method other than onlay had a severe complication. These comprised: five IPOMs, six sublay repairs, three skin graft repairs, one other procedure and one procedure unknown. Nine of these 16 complications (56%) were regarded as severe in that intensive care was needed because of respiratory failure, but there was no severe surgical complication.

**Table 4:** Surgical complications according to the Clavien Dindo classification

Clavien classification	Subgroup	Number of patients (%)
No complication	0	327 (80%)
Clavien I–IIIa	1	58 (14%)
Clavien IIIb	2	7 (2%)
Clavien IV	3	12 (3%)
Clavien V	4	4 (1%)

Clavien I–IIIA = mild to moderate complications; Clavien IIIB (surgical intervention in general anaesthesia needed), Clavien IV (admission to ICU) and Clavien V (death) = serious complications. Number of patients in each subgroup and percentage of the entire group

**Fig. 2:**
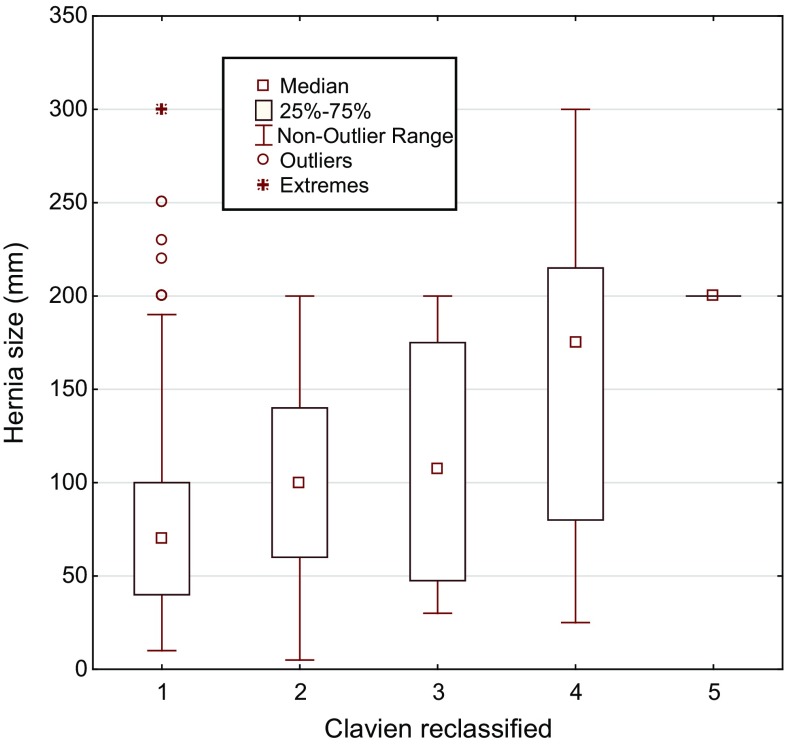
Correlation between hernia size aperture and complication graded by modified Clavien score. Patients where hernia size was recorded (*N* = 356). *p* < 0.001. 1 = no complication, 2 = Clavien 1–3a, 3 = Clavien 3b, 4 = Clavien 4, 5 = Clavien 5

Hernia aperture size and age were the only factors seen to be significant risk factors for surgical complications in both uni- and multivariate (Table 5) analyses. There was also a directly proportional relationship between hernia size and Clavien complication class when analysed with the Kendall Tau test, *p* < 0.001 (Fig. 2). BMI (*p* = 0.004) and age (*p* = 0.001) analysed as single factors were also correlated with Clavien complication class.

**Table 5:** Factors potentially influencing risk for surgical complication

Independent variable	OR	Univariate		OR	Multivariate	
		95% confidence interval	*p* value		95% confidence interval	*p* value
Onlay	2.53	1.29–4.96	0.006	1.47	0.64–3.38	0.364
Recurrence	1.23	0.68–2.23	0.494			
Concomitant bowel surgery	2.15	1.14–4.04	0.018	0.99	0.44–2.22	0.986
Hernia size (mm)	1.01	1.01–1.02	<0.001	1.01	1.00–1.01	0.006
Hernia size > median	2.35	1.36–3.98	0.002			
BMI ≥ 35 kg/m^2^	2.01	1.14–3.57	0.017	1.78	0.91–3.49	0.094
Smoker	0.93	0.39–2.22	0.872			
ASA ≥ 3	2.29	1.41–3.74	<0.001	1.65	0.92–2.96	0.093
Age	1.03	1.01–1.05	0.006	1.03	1.00–1.05	0.028
Male gender	0.82	0.50–1.34	0.432			

Odds ratio (OR) for post-operative complication after ventral hernia repair in ordinal logistic regression. Multivariate analysis included factors that increased the risk for complication in the univariate model. A *p* value ≤0.05 was considered significant. 347 patients included in the multivariate analyses. Hernia size is calculated in mm transverse

There was an association between hernia aperture size and age (*p* = 0.001; Spearman) and concomitant bowel surgery (*p* < 0.001; Kendall Tau). However, when repeating the ordinal regression analysis without these factors hernia size still manifested as the most significant determinant with the same OR and a *p* value <0.001. Correspondingly, age had an OR of 1.03 and *p* = 0.059, while concomitant bowel surgery had an OR of 1.2 and *p* = 0.23 when excluding hernia aperture size from the analysis.

Morbid obesity, onlay repair, high ASA-score (≥3) and concomitant bowel surgery all manifested as risk factors for surgical complication in the univariate, but not in the multivariate analysis. Gender was not a risk factor for complication, nor was recurrent hernia repair (Table 5).

The only risk factor for readmission was age (Table 6).

**Table 6:** Factors potentially influencing risk for readmission

Independent variable	OR	95% confidence interval	*p* value
Onlay	1.08	0.31–3.71	0.91
Recurrence	0.78	0.32–1.89	0.58
Concomitant bowel surgery	0.46	0.19–1.14	0.09
Hernia size (mm)	1.00	0.99–1.01	0.75
Hernia size > median	0.96	0.43–2.14	0.93
Obesity >29 kg/m^2^	1.07	0.51–2.24	0.85
Severe obesity ≥35 kg/m^2^	1.15	0.43–3.11	0.78
Smoker	0.90	0.26–3.10	0.86
ASA ≥ 3	0.75	0.36–1.58	0.45
Age	0.97	0.94–0.99	0.02
Male gender	1.16	0.55–2.43	0.70

Odds ratio (OR) for readmission after ventral hernia repair, univariate logistic regression analyses

## Discussion

Risk for early complications following ventral hernia repair rises with an increase in hernia aperture size. The direct relationship between hernia size and frequency and severity of surgical complications shown in the present material has not previously been described. This correlation was strong, indicating that hernia aperture size is of great importance in the preoperative risk evaluation of patients. The odds ratio of 1.01 means that the odds for a surgical complication increases by 1% for each mm increase in aperture size. This analysis shows a proportional relationship between hernia aperture size and Clavien complication class, thus telling us that there is no obvious cut-off size. One possible reason why this correlation has not previously been described is that most reports have come from retrospective studies where the size of the hernia aperture was unknown. When assessing a patient for ventral hernia repair, one must take into account hernia size, though it must be emphasised that not all ventral hernia patients need surgery and that most hernias increase in size with time [14]. Early incisional hernias grow rapidly and often involve the whole length of the incision if not repaired in time. Around 50% of incisional hernias manifest within the first 12 months [15]. Late hernias are less well described and are presumed to be result of failure of collagen in the scar [16]. Intraoperative measurement was used as determinant of the hernia aperture size. In a previous study of abdominal rectus diastasis, clinical assessment corresponded more precisely to the intraoperative measurement of the hernia than a CT scan [11]. Thus, a preoperative clinical assessment is considered to correspond to the calculated risk estimates in the present study. However, the clinician should be aware that the time span between clinical assessment or a CT scan and the time of surgery may allow for an increase in the hernia size. The fact that hernia size manifests as the solely most important risk factor for early surgical complications challenges two cornerstones in hernia treatment: watchful waiting [17] and delay of surgery in attempt to reduce weight [18]. Both these concepts must be held against the risk of an increasing hernia size. Since the natural course of early appearing incisional hernias often means extension to the entire previous incision, a prompt repair might be advocated even in patients with moderate symptoms. Watchful waiting may still be justified with a small asymptomatic hernia and high comorbidity. However, with increasing hernia size a choice must be made whether or not to apply lifelong conservative treatment. Watchful waiting concept is partly a reaction to the fact that recurrence rates with long-term follow-up is quite high and also that morbidity and mortality rates after incisional hernia repair are troublesome [19]. The same is true for obesity where a high BMI affects recurrence rates, increases infection rates and also adds a considerable risk if an emergency intervention may become necessary. Furthermore, it is well known that physical training is often embarrassed by spatial effects of the hernia per se.

To assess the most appropriate treatment principle, knowledge of the natural course of ventral hernia is important. A small incisional hernia occurring early post-laparotomy should be repaired while it is still small. These hernias almost always expand over time and extend to the entire incision leading to substantial comorbidity [14]. Current analysis states the importance of timing and suggests that prompt intervention in some cases is beneficial. Watchful waiting studies have sometimes prioritised analysing risk of acute intervention, thus neglected the risk of a more complex repair at the time of intervention [14, 20].

There are reports in the literature [21] indicating concomitant bowel surgery to be a risk factor for surgical complication. In the present study, concomitant bowel surgery manifested as a risk factor in the univariate but not in the multivariate analysis.

When performing concomitant bowel surgery, it is of utmost importance that the surgical team is experienced and well prepared. A meticulous adherence to hygienical principles: changing of gloves and instruments as well as re-sterilising the abdominal skin before inserting the mesh and use of modern large pore biomaterials of polypropylene or PVDF are principles routinely exerted by the authors. When assessing risk for adverse event in the individual case, it is necessary to take into account that concomitant surgical procedures might increase the risk for complication. However, performing the two procedures on different occasions can also increase post-operative morbidity since there will be two episodes of general anaesthesia and two post-operative courses. The reason for choosing an onlay procedure was not registered in the database. If an onlay repair is considered the most appropriate method for a specific patient, then maybe seroma should not be regarded as a complication, but rather an expected consequence of surgery.

Advanced age, several comorbidities and obesity have been associated with a higher risk for complication in previous studies [21]. In the present study, age was a risk factor for surgical complication in the multivariate analysis, but an association between obesity and complication could not be demonstrated. In the univariate analysis, obesity was associated with an increased risk, as was ASA physical score ≥3 (indication comorbidities, although not specifying which). There is a possibility that the study did not include enough patients to be able to demonstrate an existing difference regarding these variables in a multivariate analysis.

Describing risk in ventral hernia repair is a complex matter. Hernia size is associated with risk in the present study. There are, however, other important determinants, for instance, loss of domain, which was not included in our register [22]. Such analyses require special assessment of imaging material that is demanding on resources, but may be motivated in future randomised trials. Variables to be included in a register must be chosen with care [23]. If too many parameters are included or are too complicated to fill in, compliance and completeness of registration may be hampered.

Comparison of SSO rates between studies is difficult due to the fact that no adjustment of complexity is possible due to the lack of validated risk factors. Present material constitutes tertiary referral centre cases and thus expected to have a higher incidence of post-operative complications. One explanation for the relatively low complication rate (20%) in the present material, if one consider the patient selection, may have been the mandatory cessation of smoking prior to surgery practised at the two participating units [24]. For evaluation of hernia site complications, longer follow-up than 3 months is necessary. This is in order to see complications such as recurrence, pseudo-hernia and mesh migration. In contrast to earlier recommendations, synthetic material was used in cases with concomitant bowel surgery. No obvious increase in risk for SSO was detected by this practice. Modern management of complex ventral hernia repair requires a dedicated team that, after multimodal assessment and optimisation of identifiable health problems, can tailor the surgical approach to match the patient’s function and physical activity. The onlay technique is a valuable method when repairing a complex ventral hernia where other techniques are not feasible or inferior due to complicated anatomical conditions. In a previous study, we found that functional outcome in terms of abdominal wall muscle strength does not differ between the techniques used for reconstruction [25], although cases operated with onlay repair were more often re-repairs. In complicated cases, the higher risk for SSO and seroma associated with the onlay technique may be considered acceptable. Such cases include major concomitant bowel surgery where maximal distance between mesh and viscera is desirable, re-recurrence where previous mesh insertion obliterates the sublay space, and cases with anticipated dense adhesions which make the IPOM position unsuitable. The presence of excessive scar tissue and previously implanted mesh has a substantial impact on tissue remodelling and repair, and predispose to healing disturbances that increase the risk for surgical complications [26].

The most common severe complication in our study was respiratory failure. None of the respiratory failures reported were induced by sepsis. The respiratory failures reported were considered due to physiological shifts related to reduction in large hernias where the return of hernia contents into the abdominal cavity increased the respiratory demands considerably, even though the patient had worn a tight abdominal binder for several months prior to surgery. We propose that there appears to be an association between large hernia aperture and respiratory failure leading to unplanned ICU care. The aperture size should always be assessed as part of the preoperative management of the ventral hernia patient. In cases where the hernia is large, preoperative investigation and optimisation of the patient’s respiratory function are essential in order to reduce the risk for respiratory complications. In summary, according to the present findings, the larger the hernia, the greater the risk for early surgical complications, including significant complications such as respiratory decompensation. As hernias often grow in size over time, delaying repair may result in a larger hernia to repair and therefore greater risk for complications. Thus, the risks of waiting for improvements in a patient’s condition, such as waiting for weight loss, should be balanced against the risks related to possible growth in the hernia over time.
